# A Mental Wealth perspective: crossing disciplines to understand the value of collective mental and social assets in the post-COVID-19 era

**DOI:** 10.1186/s13033-022-00568-1

**Published:** 2022-12-12

**Authors:** Kristen Tran, John Buchanan, Yun Ju Christine Song, Sebastian Rosenberg, Jo-An Occhipinti, Ian B. Hickie

**Affiliations:** 1 Brain and Mind Centre, Faculty of Medicine and Health, University of Sydney, Camperdown, Australia; 2 Mental Wealth Initiative, University of Sydney, Camperdown, Australia; 3 Sydney Business School, University of Sydney, Sydney, Australia; 4 Computer Simulation & Advanced Research Technologies (CSART), Sydney, Australia

**Keywords:** Mental capital, Wellbeing, Mental assets, Social capital, Mental health, Health policy

## Abstract

**Background:**

A reconceptualised global strategy is key as nations begin to shift from crisis management to medium- and long-term planning to rebuild and strengthen their economic, social and public health systems. Efforts towards measuring, modelling, and forecasting Mental Wealth could serve as the catalyst for this reconceptualization. The Mental Wealth approach builds systemic resilience through investments which promote collective cognitive and emotional wellbeing. This paper presents the theoretical foundations for Mental Wealth. It presents, for the first time, literature across the disciplines of health and social sciences, economics, business, and humanities to underpin the development of an operational metric of Mental Wealth.

**Discussion:**

An approach which embeds social and psychological dimensions of prosperity, alongside the economic, is needed to inform the effective allocation of investments in the post-pandemic world. The authors advocate for a transdisciplinary framework of Mental Wealth to be applied in innovating population-level policy interventions to address the growing challenges brought on by COVID-19. Mental Wealth highlights the value generated by the deployment of collective mental assets and supporting social infrastructure. In order to inform this position, a review of the literature on the concepts underpinning Mental Wealth is presented, limitations of current measurement tools of mental and social resources are evaluated, and a framework for development of a Mental Wealth metric is proposed.

**Conclusion:**

There are challenges in developing an operational Mental Wealth metric. The breadth of conceptual foundations to be considered is extensive, and there may be a lack of agreement on the appropriate tools for its measurement. While variability across current measurement approaches in social resources, wellbeing and mental assets contributes to the difficulty creating a holistic and generic metric, these variations are now clearer. The operationalisation of the Mental Wealth metric will require comprehensive mapping of the elements to be included against the data available.

## Background

The ongoing emergence of new variants of SARS-CoV-2 indicate that COVID-19 is here to stay. Its continued grip has prolonged economic disruptions, hindering nations in their path to recovery. A reconceptualised global strategy is key as nations begin to shift from crisis management to medium- and long-term planning to rebuild and strengthen their economic, social and public health systems. As nations prepare for an endemic stage of COVID-19 alongside continued global challenges such as food insecurity and climate change, a systems approach based on resilience is crucial to prepare economic, social, and health systems for future shocks [1]. COVID-19 has exposed and exacerbated pre-existing systemic challenges in healthcare [2], income inequality [3, 4] and social vulnerability [5]. An approach which embeds social, psychological dimensions alongside the economic are needed to inform the effective allocation of investments in the post-pandemic world.

This article discusses the need for a Mental Wealth perspective in reconceptualising economic growth and building systemic resiliency. It argues the importance of developing a meaningful population-based measure for Mental Wealth to facilitate accountability amongst governments, for monitoring and to guide continued investment. The Mental Wealth metric gives pre-eminence to the collective social and mental assets within the population, which underpins a nation’s resiliency and contributes to its prosperity. It gives importance to the interrelationships between social, commercial, and structural determinants of mental health and wellbeing. Both mental health and wellbeing play crucial roles in mediating the ability to acquire and deploy mental capital for community development. It empirically challenges the idea that strengthening universal social prosperity is incompatible with economic and commercial interests. A Mental Wealth metric requires consolidating disparate evaluation and measurement tools from the health and social sciences, economics, business, and the humanities disciplines. Mental Wealth’s transdisciplinary framework and use of modelling techniques rooted in complex systems science aims to address the limitations of existing population measures of mental and social assets, and to give transparency in the systems by which they are determined.

The concept of ‘Mental Wealth’ originated from The Foresight Project on Mental Capital and Wellbeing, by the UK Government Office for Science [6]. It was a landmark study giving insights on the development of mental capital and mental wellbeing across the life course, and the potential opportunities and challenges nations faced in fostering these dimensions [7]. Mental Capital was defined as ‘the totality of an individual’s cognitive and emotional resource’ and Mental Wellbeing as ‘a dynamic state in which the individual is able to develop their potential, work productively and creatively, build strong and positive relationships with others, and contribute to their community’ [6]. The two concepts jointly form the foundation of Mental Wealth, where the state of Mental Wellbeing plays a critical role in determining how Mental Capital is developed and deployed. This initial work provided extensive insight into the pathways in which mental capital grows and declines over the individual’s life course, highlighting the opportunities governments and others have in generating environments for mental capital and wellbeing to prosper.

The Mental Wealth Initiative (MWI) of the University of Sydney’s Brain and Mind Centre in partnership with the Sydney Business School is a multi-faculty enterprise, working with external leaders across academia, government, business, law, mental health and social policy, and communities [8]. The MWI seeks to build on the original work by Beddington et al., to identify and promote policy opportunities to foster Mental Wealth by measuring, modelling, and forecasting the Mental Wealth of nations [7]. The MWI expands on the original definition of Mental Wealth, by defining it as ‘*a measure of national prosperity that captures the value generated by the deployment of collective mental assets and supporting social infrastructure’*[9]. One of the first priorities of the MWI is to shift the ‘boundary of production’ that informs what is valued within systems of national accounts; moving beyond Gross Domestic Product (GDP) to include activity that strengthens the fabric of society. The Mental Wealth metric employs a non-market valuation method to value social contributions (the goods and services delivered where no financial remuneration is received, it includes volunteering, unpaid care, civil participation, etc.) as well as government and non-government expenditure on the social infrastructure that supports social productivity, not already captured by GDP. National Mental Wealth is the monetary value of social contributions and social infrastructure investment, additive to a downwardly adjusted real GDP over a given period [9]. This requires a study of the interactions between mental health, economic and social systems. Therefore, analytic methods of complex systems science; namely, systems modelling and simulation of Mental Wealth will be undertaken to understand these interactions, and to consider which policy-mediated changes in economic, social, and health sectors could enhance this new metric of national prosperity. An open-access, interactive simulation model of Mental Wealth would facilitate greater transparency in the decision-making process, allowing assessment of investment decision trade-offs given alternative intervention strategies and timeframes [10] and help build consensus for collective action by regional decision-makers [11].

The MWI’s monetization approach to measuring Mental Wealth, is a distinct shift from other efforts which have aimed to reconceptualise the meaning of a prosperous society, for example the Wellbeing Economy [12] and Green Economy [13], and those which employ an index-based approach consisting of weighted indicators generating a composite score of prosperity [14]. These previous developments have enriched our understanding of wellbeing and living standards beyond what the traditional metric of GDP has been able to convey. Though these index-based approaches do have their limitations. Occhipinti et al. [9] have highlighted two main issues, first being the difficulty in assessing overall progress of composite indexes, where movement in individual indicators (and the cases of movement in opposing directions) may not be reflected and fully communicated in a composite measure. Secondly, indices fall short in acknowledging the causal interrelationships between the indicators they measure. They provide a list of factors contributing to prosperity, without a systems analysis which would aid in interpreting which factors should be prioritised to deliver the most beneficial outcome to national prosperity at a given point in time. The monetization approach to measuring Mental Wealth has an advantage in its ease of interpretability to policy makers, who have traditionally been receptive to other monetised metrics, namely GDP.

Across all nations, mid- to long-term policy strategies are needed to address the mental health consequences of the pandemic. The adverse effect on population mental health from past economic downturns have been analysed at length, including the 2008–2009 global financial crisis [15–17]. Continual and time-delayed negative impacts on population mental health, including suicidal behaviour, has been associated with economic shocks [18]. This should serve as a warning to governments to proactively remedy the time-delayed consequences of the COVID-19 pandemic. The pandemic has been associated with substantial mental health deterioration revealing social inequalities. While adverse mental health impacts have increased as a result of the social and economic impacts of the pandemic, social protection measures have acted to mitigate the full extent of the negative impact [19]. With the rapid and significant stimulus and relief efforts deployed by the International Monetary Fund, the World Bank Group and individual governments, many nations were able to stave off immediate unemployment and economic hardship for some, but such measures are typically time limited. Governments have considerable scope to realise more wholly a deliberate and long-term strategy to strengthen the socioeconomic systems and sustainably reduce social vulnerability. Large scale and effective interventions directed at the social aspects of life, which work towards supporting the mental health and wellbeing of populations, is necessary to have greater resiliency to the consequences of crises such as the COVID-19 pandemic.

## Discussion

Mental Wealth and its drivers have been founded on the large body of research that supports the need to address the social and economic determinants of health. Poor social and economic circumstances negatively impact health throughout the life course, in every society. The social gradient in health shows lower socioeconomic position equates to at least twice the risk of serious illness and premature death [20]. A systematic review of 115 studies conducted on epidemiological literature of common mental disorders and poverty in low and middle-income countries showed over 70% found positive associations between a range of poverty measures and common mental disorders [21] whilst a review of European population surveys showed reporting of higher frequencies of common mental disorders are associated with material disadvantage, unemployment, and low educational attainment [22]. The life course has a series of critical transitions and can be thought of in terms of a trajectory, where each stage can affect health by propelling people onto a path of greater advantage or disadvantage. Beddington and colleagues’ concept of mental capital trajectory brings to attention the various factors that can support or undermine the accrual of mental capital at each stage of life; rising at early life, plateauing during the middle, and declining in the later years due to growing vulnerability to disease and age-related changes [23]. Action through policy interventions throughout these life stages can impact the individual’s life course and improve population mental health, reducing risk of the mental disorders that are associated at each life stage and addressing social inequalities. Focusing on growing Mental Wealth in strategies to promote national prosperity prioritises the need to address the social and economic inequities that impact mental health and mental capital accumulation.

### The framework for collective mental assets

A framework for collective mental assets provides a lens for policy makers to engage in decisions that are structural and can be assessed at the population level. This integrates a greater awareness towards the community level movements that ultimately influence the accrual of mental assets and social capital. These movements generate the macro-social contexts that influences and supports individual level behavior change. Defining the components mental capital and mental health and wellbeing as a nation’s collective assets (rather than assets belonging solely to the individual) can facilitate a mechanism to hold governments accountable for ensuring individual, community and macro-economic and -social growth and its continued investment.

#### Mental capital

The concept of Mental Capital encompasses a person’s cognitive and emotional resources. It includes their *cognitive capability, learning efficiency*, and their *emotional intelligence* [7]. Though there is a vast body of literature relating to the individual components of Beddington’s concept of mental capital, these have mainly focused on studying the outcomes and abilities of individuals.

*Cognitive ability* can be defined as a general mental capability relating to ‘reasoning, problem solving, planning, abstract thinking, complex idea comprehension, and learning from experience’ [23]. General Mental Ability (GMA), often used as an indicator of cognitive ability, has shown to be predictive of occupational status, job performance and job complexity and an important indicator for quality of life [24].

*Learning efficiency* is a measure of improvement in performance accuracy and speed per amount of learning time [25] and can be thought of in conjunction with skill learning “a change, typically an improvement, in perceptual, cognitive, or motor performance that comes about as a result of training that persists for several weeks or months” [26].

*Emotional intelligence* is the “capacity for recognizing our own feelings and those of others, for motivating ourselves, and for managing emotions well in ourselves and in our relationships” [27]. It also encompasses our social skills and stress resilience.

Beddington and colleagues sought to explain the links between a vast set of biological, social and economic factors and the trajectory of mental capital of an individual’s life span. The MWI seeks to recognise the value of a nation’s cognitive and emotional resources as more than simply the sum of each individual’s assets but to capture the value in their deployment to achieve collective outcomes [9]. The measurement and formation of the conceptual model of collective mental assets will require drawing insights and building upon the prevailing related models, such as *human capital, psychological capital, brain capital* and the mental wellbeing concept.

*Human capital* encompasses the attributes of a population that contribute to economic productivity, alongside physical capital such as buildings, and other tangible assets [28]. The OECD [29] defines human capital as the knowledge, skills, competencies and attributes embodied in individuals that facilitate the creation of personal, social and economic wellbeing. Numerous studies have examined the association between a range of dimensions of human capital and economic growth and productivity [30–33]. The World Bank has brought this to the forefront, highlighting current health and education outcomes as key determinants of productivity in the next generation of workers, through its Human Capital Project [34]. The Project aimed to “understand the link between investing in people and economic growth, and to accelerate financing for human capital investments.” Monitoring the expected formation of human capital in the next generation, as a measure of the effect of near-term investments in health and education, can work to grow accountability amongst nations to their people in making these investments [35]. The contribution of the Human Capital Project has been pivotal in the reconfiguring of how healthcare is understood and planned, linking healthcare strategy to GDP growth.

Arguments have also been mounted against adopting the notions of human capital theory in public policy. It raises concerns in a few domains, particularly in healthcare equity and the model of education, by placing an arguably imbalanced attention towards those who are formally economically productive. It creates a situation where there is exclusion of those who are traditionally not defined as participants in the labour market, those with disabilities, chronically ill and unable to work [36]. The human capital theory application to education policy has highlighted the large effect of acquiring knowledge and skills on a person’s income-generating abilities. Simultaneously it has imposed a damaging goal orientation in education policy by promoting an instrumental view of education, where the value of education and skills are only of value to the extent in which they contribute to boosting productivity and higher wages [37]. Many argue this interpretation of education, as a means to accumulate human capital, is impoverished and instead seek to evaluate education as playing a role in an individual’s capability to achieve broader valued functioning [38]. Wigley and Akkoyunlu-Wigley [39] found that educational attainment had a significant effect on life expectancy independent of its effect by way of increased income. Empirically showing how income undervalues the health functioning achieved by educational attainment, supporting that education should be valued in terms of capability for functioning, as opposed to a resource for economic productivity. The Mental Wealth approach seeks to account for the value of knowledge, skills and competencies as greater than their ability to produce economic outputs, valuing also their ability to enhance social productivity and wellbeing.

Building on the human capital concept, the term ‘*Brain Capital*’ was coined in recognition of the need for economic reimagination, incorporating brain health and brain skills as contributors to a ‘Brain Economy’ [40]. There is mounting evidence that unless nations strengthen their human capital, they will be unable to achieve sustained, inclusive economic growth. Preparing the workforce for the demands of more highly skilled jobs in the future is required in order to be able to compete in the global economy [41]. In this light, Smith et al. has brought attention to the necessity of Brain Capital in this new era of the ‘Brain Economy’, where ‘most new jobs demand cognitive, emotional, and social, not manual, skills and where innovation is a tangible “deliverable” of employee productivity’ [40].

The policy agendas born from human capital theory and its measurement, such as the Human Capital Index [42], has some merits in growth-based policies but has limitations for use in guiding overall health and education policy. A broader development discourse is needed. One which incorporates a wider metric that captures the value of cognitive and emotional resources that are deployed and contribute to society, contributions which are not captured by traditional economic growth indicators.

#### Mental wellbeing

Mental wellbeing can be viewed as conditioning an individual’s ability to work productively and develop their potential, determining their ability to attain and deploy mental capital [6]. A key finding of the Foresight Project is that mental wellbeing and mental capital are intertwined. Intentions to affect the growth of mental capital require fostering an environment which enables a high level of collective mental wellbeing [6]. To achieve change, it is essential to understand the epidemiology of mental wellbeing across the nation, at the same time recognising variation by region and across the metropolitan and regional divide.

Mental wellbeing is an important but understudied idea at the population level. This is partially due to the lack of suitable population-based measures. Development of instruments to quantify and monitor mental wellbeing at a population level is key to the evaluation of mental health promotion initiatives and to the understanding of mental capital accumulation and deployment. Definitions of wellbeing are notoriously broad, often interwoven with related concepts such as, life satisfaction, positive mental health, quality of life, happiness, mental capital and human functioning [43]. A more clearly defined and multifaceted measure of mental wellbeing is needed to enhance studies in the appraisal of the diverse health and functional states. Current measures of mental wellbeing fall into three categories: Evaluative measures (life satisfaction), Eudaimonic measures (meaning in life), Hedonic measures (positive or negative affect) [44]. Mental wellbeing has been conceptualized as a concise psychological construct incorporating psychological functioning, alongside the cognitive and emotional aspects of wellbeing. Though current available measures are pre-occupied with states of subjective experience, appearing not to account for the setting or regimes in which they are embedded which can largely influence its state.

In this current climate of instability, where COVID-19 has impacted people’s lives and wellbeing in fundamental ways, personal positive resources are needed. *Psychological Capital* is a construct said to safeguard people’s capacity to successfully maintain their mental health, by increasing their well-being and decreasing their levels of anxiety and/or depression [45]. Incorporation of the elements of Psychological Capital in a mental asset measurement allows for the accounting of a resource required for collective social and stress resilience. There are four main psychological resources from the positive psychology literature that form the higher-order construct of Psychological Capital: self-efficacy, hope, optimism and resilience [46]. A Psychological Capital lens, drawing from positive psychology concepts and organisation behaviour theory, could aid in accounting for the emotional resources and functions of collective mental assets.

A number of mental wellbeing metrics have been developed including The Warwick-Edinburgh Mental Well-being Scale, which was created to meet the demands for a population level measure [47]. It synthesised the two main camps of wellbeing research, hedonic and eudaimonic, in a short and meaningful measure that has shown to have a psychometrically robust scale, with no ceiling effects in a population sample offering a promising evaluation tool in research and public health practice. Further research and development of robust mental wellbeing metrics such as this is key in understanding the trends and influences of mental wellbeing nationally. In addition, greater emphasis is needed on measuring collective rather than individual subjective wellbeing.

### Capturing social productivity

The MWI extends the original definition of Mental Wealth to place emphasis on the value of social infrastructures, aiming to recognise the value of a society’s collective capabilities as greater than the sum of individual actions. It attempts to account for non-market value generated from social cohesion and participation, and civic engagement. There is growing consensus that social capital including norms, networks and trust can work to contribute to accomplishing critical tasks in emergency situations [48]. This is of high relevance and importance in the current climate. It has been found that social capital stemming from greater trust and relationships within a community, can facilitate calm and rapid collective action in a crisis context [49, 50]. It was found during the COVID-19 pandemic in the United States, that moving a county from the 25th to the 75th percentile of the distribution of social capital would lead to a 18% and 5.7% decline in the cumulative number of infections and deaths [51]. Social resilience and collective collaboration can function to aid communities to rebuild and recover in national emergency situations.

Putnam’s [52] notion of social capital focuses on features of social life that empower individuals to act together more effectively to pursue shared objectives. Viewing networks of civic engagement (e.g., neighbourhood associations, sports clubs, volunteer groups) as a vital form of social capital that fosters strong notions of reciprocity that in turn propels social trust. Social capital incorporates the resources that exist in the links between people and networks, which differs from human capital; the stock of expertise amassed by an individual. It is essential to the production and preservation of collective well-being [53]. Active community involvement, specifically, voluntary work is widely recognised as beneficial for mental wellbeing. Community involvement is a widely cited suggestion in policy guidelines for active ageing in Australia, New Zealand and United Kingdom reflecting The World Health Organization recommendation for the elderly as they transition out of structured employment environments [54].

Contrasting with traditional health psychology which centres on individual decisions as drivers of health behaviours, community health promotion approaches incorporate the notion of social norms alongside individual decisions in driving health related behaviours. Recognising that most people are more likely to alter their high-risk health behaviours when in situations where they observe their trusted and liked peers in their community changing their behaviours [55]. The complex social processes within the community have been evaluated and operationalised by social theorists as one way of understanding how the formation of social relations has produced resources that underpin population health. Social capital is thought to support population health through the two pathways of social trust and networks of reciprocity, fostering greater cooperation and enabling collective action in the face of challenges [56]. Many studies have shown an association between higher social capital with better self-rated health at the aggregate or individual level [57–59]. Though due to the wide variation of health outcomes and social capital indicators used to study this relationship, it is difficult to ascertain which key mechanisms and dimensions of social capital drive positive outcomes in individual and community health [60].

Findings in the literature suggest that social capital also has an impact on economic growth which is at least as strong as that of human capital or education. Social capital contributes to economic development [61] and GDP [62], by improving the return on investment in physical and human capital. Social capital impacts growth by supporting accumulation of human capital, through financial development influenced by collective trust and increased networking between firms, both of which lead to the production and diffusion of technological innovations and business activity. The interpersonal trust component of social capital has been shown to have an important role in explaining the efficiency of political institutions, and in the economic performance of contemporary societies [63]. Civic engagement has also been found to be an independent determinant of economic growth in U.S. counties, where per-capita income grew more rapidly in counties with high levels of social capital (measured using the density of membership organizations, charitable giving and voter participation) [64]. Greater attention by governments in building social capital is needed, as the stream of benefits from accumulated stock often enhances output through raising the productivity of the other heavily invested resources in the economy, physical and human capital.

#### Investing in social capital to enhance social productivity

Akin to other forms of capital, social capital is not costless to produce, requiring substantial investment in time and resources. According to Bourdieu [65] social capital is ‘the product of investment strategies, individual or collective, consciously or unconsciously aimed at establishing or reproducing social relationships that are directly usable in the short or long term’. Given the economic and societal contributions of social capital, a commitment to social capital building and investment should be a main fixture in national strategies to enhance social prosperity. Though early references to social capital formation suggested it was a resource that was produced by long-term socioeconomic progress, independent of public policy, recent studies indicate that government policies have major effects [66]. It was argued that cross-country divergences in level of social capital stock could be explained by the level of government commitment to facilitate social capital formation and action from governments could reduce social capital poverty traps, resultant of the coordination problem between private agents in an economy [67]. Development of an investment evaluation tool able to quantify the contribution of social capital and its deployment, Social Productivity, can aid governments in their policy evaluations by communicating its investment return.

#### Measuring social capital

Despite the relevance of social capital, few attempts have been made to quantify the effects of social capital. Discussion on the conceptualisation of social capital has evolved further than the efforts towards the development of a standardised set of tools for its empirical measurement. Fine [68] describes the concept as slippery and deceptively simple; despite the volume of social capital research a clear and uncontested definition and theory has yet to be reached. Definitions and theories of social capital do provide a starting point for the development of conceptually sound measures, and advances in research on social capital generally examine the two main types of social capital: bridging (between groups) or bonding (between individuals in a group). Within these types there are structural, cognitive and relational components which operate at the macro or micro level. Much of the social capital research to date are studies which have drawn conclusions and outcomes from secondary analyses, often involving a mixture of measures and indicators which contribute to the lack of clarity between the social capital theory and its measurement. There are a few studies using primary data collection, expanding what has been developed through secondary analysis. There are numerous social capital indicators that have been developed, which aim to capture the dimensions of social capital and are amenable to qualitative and quantitative study, and generally evaluated in tandem. However, there is difficulty in fully capturing the underlying structures of social capital and distinguishing between the effect at the individual and the community level. Hawe and Shiell [69] suggest that the research on social capital in relation to health has been susceptible to this difficulty, in capturing and distinguishing between the contextual level influences as well as the individual level influences. Though studies which have incorporated a multi-level structure in their analysis of individuals within their social units have been an important development in social capital research [70].

Methodological diversity is needed to appropriately reflect the context dependent qualities of social capital. This has shown to be an obstacle in aggregating and scaling upwards in studies as well as having the ability to compare and build theory across different studies [71]. One approach is looking to obtain a monetary value of social capital, often estimated using a well-being function. Other monetization approaches being explored include the shadow price approach (as a form of compensating variation), stated preference techniques, and hedonic approaches [72–74]. Orlowski and Wicker [75] undertook an empirical examination based on data from the European Values Survey covering 45 European countries, applying a four-dimension conceptualization of Social Capital. They examined the monetary value which could be assigned to interpersonal trust, institutional trust, trustworthiness, and participation in civil society using standardized shadow prices. This allowed for comparisons between the different social capital indicators, and findings indicated that social capital has significant monetary value to individuals. Development of a multi-method approach is needed to reveal the mechanisms in which social capital contributes to the economy and its association with collective mental assets. In reference to mental health research within social movements and capital, Campbell [76] emphasizes the need for awareness of power and inequity as central to community mobilisation planning. Requiring intersectional approaches that highlight how population subgroups are being oppressed, as social capital research has generally focused on the benefits to dominant groups in society. There is still a need for awareness of the contexts in which social capital resides and the forces of other determinants and intersectional identities when evaluating mental health concepts and conducting social capital research.

## Conclusion

Innovation in public mental health interventions is needed to address the growing challenges brought on by COVID-19. The development of an operational Mental Wealth metric is key in orienting governments to place greater recognition and value on mental and social assets that underpin healthy and prosperous societies. There are challenges in developing such a metric. The conceptual framework of the MWI has evolved from decades of prior research in mental health and development and wellbeing. The breadth of conceptual foundations to be considered is extensive, and there may be a lack of agreement on the appropriate tools for its measurement. Variations between current measurement approaches for social resources, mental wellbeing and mental assets contribute to the difficulty of creating a holistic and generic metric. We have described current variation in the definitions and measurements of the pillars of Mental Wealth. The operationalisation of the Mental Wealth metric will require comprehensive mapping of the elements to be included against the data available. This is the subject of further research and debate. The adoption of a Mental Wealth perspective in reconceptualising economic growth and rebuilding economic, social, and public health systems should be a priority to aid post-pandemic reconstruction. Population-level policy interventions that focus on strengthening collective mental assets and social infrastructures can drive economic and social benefits and boost national Mental Wealth.

## Data Availability

Not applicable.

## Abbreviations

BMC: Brain and Mind Centre
MWI: Mental Wealth Initiative
GDP: Gross Domestic Product
GMA: General Mental Ability
